# Characteristics of Hospital-Acquired and Community-Onset Blood Stream Infections, South-East Austria

**DOI:** 10.1371/journal.pone.0104702

**Published:** 2014-08-08

**Authors:** Martin Hoenigl, Jasmin Wagner, Reinhard B. Raggam, Florian Prueller, Juergen Prattes, Susanne Eigl, Eva Leitner, Katharina Hönigl, Thomas Valentin, Ines Zollner-Schwetz, Andrea J. Grisold, Robert Krause

**Affiliations:** 1 Section of Infectious Diseases and Tropical Medicine, Medical University of Graz, Graz, Austria; 2 Division of Pulmonology, Medical University of Graz, Graz, Austria; 3 Division of Infectious Diseases, University of California San Diego, San Diego, California, United States of America; 4 Clinical Institute of Medical and Chemical Laboratory Diagnostics, Medical University of Graz, Graz, Austria; 5 Institute of Hygiene, Microbiology and Environmental Medicine, Medical University of Graz, Graz, Austria; National Institute of Allergy and Infectious Diseases, United States of America

## Abstract

**Purpose:**

The objective of this study was to compare epidemiology, causative pathogens, outcome, and levels of laboratory markers of inflammation of community-onset (i.e. community-acquired and healthcare-associated) and hospital-acquired bloodstream infection (BSI) in South-East Austria.

**Methods:**

In this prospective cohort study, 672 patients fulfilling criteria of systemic inflammatory response syndrome with positive peripheral blood cultures (277 community-onset [192 community-acquired, 85 healthcare-associated BSI], 395 hospital-acquired) were enrolled at the Medical University of Graz, Austria from 2011 throughout 2012. Clinical, microbiological, demographic as well as outcome and laboratory data was collected.

**Results:**

*Escherichia coli* followed by *Staphylococcus aureus* were the most frequently isolated pathogens. While *Streptococcus* spp. and *Escherichia coli* were isolated more frequently in patients with community-onset BSI, *Enterococcus* spp., *Candida* spp., *Pseudomonas* spp., *Enterobacter* spp., and coagulase-negative staphylococci were isolated more frequently among those with hospital-acquired BSI. With regard to the outcome, 30-day (82/395 vs. 31/277; p = 0.001) and 90-day mortality (106/395 vs. 35/277; p<0.001) was significantly higher among patients with hospital-acquired BSI even though these patients were significantly younger. Also, hospital-acquired BSI remained a significant predictor of mortality in multivariable analysis. At the time the blood cultures were drawn, patients with community-onset BSI had significantly higher leukocyte counts, neutrophil-leucocyte ratios as well as C-reactive protein, procalcitonin, interleukin-6 and serum creatinine levels when compared to those with hospital-acquired BSI. Patients with healthcare-associated BSI presented with significantly higher PCT and creatinine levels than those with community-acquired BSI.

**Conclusions:**

Hospital-acquired BSI was associated with significantly higher 30- and 90-day mortality rates. Hospital-acquired BSI therefore poses an important target for the most aggressive strategies for prevention and infection control.

## Introduction

Despite advances in therapy and supportive care, bloodstream infection (BSI) still represents a major cause of morbidity and mortality [1]–[9] in patients presenting with systemic inflammatory response syndrome (SIRS). In a population-based study the incidence of BSI was found to be 15.7/100 000 patients per year [10]. Estimations suggest that more than 23.000 people died of *Staphylococcus aureus* and *Escherichia coli* bacteremias in Europe in 2007 [11]. In Europe, incidence rates of BSIs caused by the five major pathogens (i.e. *Staphylococcus aureus*, *Escherichia coli*, *Streptococcus pneumoniae*, *Enterococcus faecalis* and *Enterococcus faecium*) have been increasing. De Kraker and colleagues reported that BSI incidence increased from 0.58/1000 patient-days in 2002 to 0.90/1000 patient-days in 2008 (7.2% per year; 95% CI 6.9–7.5%). The main increase was in *E. coli* and *Enterococcus* spp. BSI [12].

Early diagnosis and appropriate treatment of BSI may improve survival [13]. Accurate and timely diagnosis of BSI remains, however, challenging to both clinicians and laboratories. Current laboratory biomarkers are limited by either poor sensitivity or poor specificity [14], [15]. Some biomarkers are used for additional assessment, but none of them is able to replace time-consuming blood cultures. Since detection of causative pathogens and testing of their susceptibility to anti-infective drugs are essential for targeting therapy, blood cultures are the gold standard for BSI diagnosis. Still, first blood culture results are not obtained before 24 hours (at average 33 hours) [16].

Traditionally, BSI had been classified into community-onset- and hospital-acquired (HA)-BSI, as causative pathogens, resistance patterns, characteristics, and outcome had been reported to differ markedly between these two entities [17]. Due to increasing numbers of clinical outpatient treatments (e.g. patients undergoing hemodialysis) community-onset BSI has been further subdivided into healthcare-associated (HCA)- and community-acquired (CA)-BSI. This new classification, however, has been evaluated in a few studies only. Whether the differentiation between HCA- and CA-BSI is actually beneficial still remains a matter of debate.

The goal of this study was to describe the epidemiology of CA-, HCA-, and HA-BSI in a single 1500-bed university hospital. While most epidemiological studies focused on HA-BSI or community-onset BSI alone, the purpose of this study was to compare epidemiology, causative pathogens, outcome, and levels of laboratory markers of inflammation in community-onset (i.e. CA-, HCA-) and HA-BSI.

## Materials and Methods

The authors assert that all procedures contributing to this work comply with the ethical standards of the relevant national and institutional committees on human experimentation and with the Helsinki Declaration of 1975, as revised in 2008. The study protocol was approved by the local ethics committee, Medical University Graz, Austria (EC-number 21-469 ex 09/10). All participants provided their written informed consent to participate in this study once the blood cultures turned positive. For patients unable to give informed consent immediately (i.e. those requiring ICU treatment) consent was waived initially and (if possible, i.e. the patient survived) obtained later. The ethics committee approved this consent procedure.

Between January 2011 and December 2012 a total of 672 patients fulfilling criteria of SIRS with positive peripheral blood cultures were enrolled in this prospective cohort study at the University Hospital (1500 beds) of the Medical University of Graz, Austria. SIRS was defined as an acute host reaction to various different infectious and non-infectious stimuli. This definition is based on physiological parameters including body temperature (i.e. <36°C or >38°C), heart beat rate (>90 beats per minute), respiration rate (>20 breaths per minute) or oxygen saturation (paCO_2_ under 32 mmHg), as well as abnormalities in leukocyte counts (white blood cell count [WBC] over 12000/mm^3^ or under 4000/mm^3^ or over 10% immature [band] neutrophils) [18].

Before initiation of antimicrobial therapy three sets of blood cultures (i.e. three bottles BACTEC Plus Aerobic/F and three bottles Anaerobic/F) were collected (drawn from a peripheral vein, using aseptic technique). According to the manufacturer’s instruction, resins have been incorporated into BACTEC culture media of BACTEC Plus Aerobic/F and Anaerobic/F bottles to enhance recovery of organisms without the need of special processing. Blood culture sets were incubated in the continuously monitored blood culture instrument BD BACTE FX (Becton Dickinson, Cockeysville, MD) for up to seven days (if remaining negative) as described previously [14]. Each blood culture bottle contains a chemical sensor for detection of increasing CO_2_ levels produced by the growth of microorganisms. This sensor is monitored by the above mentioned instrument every 10 minutes for an increase in its fluorescence, which is proportional to the amount of CO_2_ present. A positive reading indicates the presumptive presence of viable microorganisms in the vial. Detection is limited to microorganisms that grow in a particular type of medium. If coagulase-negative staphylococci (CoNS) or gram-positive rods were detected, two or more positive blood culture bottles growing the same organism (same species, same resistance pattern) were required for a case to count as bacteremia; cases that did not meet these requirements were excluded due to likely contamination [19]. For the remaining bacterial/fungal pathogens one positive bottle was defined as sufficient evidence of bacteremia/fungemia. Blood samples for determination of laboratory infection markers were collected simultaneously with the blood cultures. Measurements were performed on an ELISA platform reader (Flex Station 3, Molecular Devices, Munich, Germany) at the Clinical Institute of Medical and Chemical Laboratory Diagnostics, Medical University Graz. C-reactive protein (CRP), procalcitonin (PCT), and interleukin-6 (IL-6) were routinely determined on a fully automated analyzer (Cobas 8000 system, Roche Diagnostics, Rotkreuz, Switzerland). Neutrophil-leukocyte ratio (NLCR) was retrospectively calculated.

Clinical data was collected at the time of study enrolment and after hospital discharge from the individual medical chart as well as the electronic hospital patient’s database. Episodes were classified as HA if the episode occurred more than 48 hours after admission [17]. All other episodes were considered community-onset BSI and further sub-classified as HCA or CA. BSI was defined as HCA-BSI when the case fulfilled one or more of the following criteria: (1) visit to a hospital or hemodialysis clinic within the past 30 days before BSI onset; (2) hospitalization for two days within the past 90 days before onset; (3) residence in a nursing home or long-term care facility [20]. All other cases of community-onset BSI were classified as CA-BSI. As the debate whether HCA-BSI should be included in community-onset BSI or analyzed separately is still ongoing, HCA-BSI was analyzed in both ways (included in community-onset BSI and analyzed separately).

Statistical analysis was performed using SPSS, version 20 (IBM, USA). Continuous data are presented as medians (inter-quartile ranges [IQR]). Patient groups were compared using Mann-Whitney U test. Univariate and multivariable logistic regression analysis were performed and odds ratios (OR) with 95% confidence intervals (CI) displayed. In a first step univariate logistic regression analyses were performed. We included explanatory variables with p<0.20 in the multivariable logistic models. Variables in the final model were selected with a forward stepwise procedure.

## Results

277 of the included 672 patients were assigned to the community-onset BSI group. A proportion (85/277 patients) was assigned to the subgroup of HCA-BSI, the remaining 192 patients to CA-BSI. Another 395 patients had been hospitalized for at least 48 hours at the time the positive blood cultures were collected. These patients were therefore assigned to the HA-BSI group.

Basic demographic data are depicted in Table 1. Patients with HA-BSI were significantly younger than those with community-onset BSI. No difference for age was found between CA- and HCA-BSI.

**Table 1:** Demographic data, duration of hospitalization and laboratory parameters of BSI cases.

	Community-acquiredBSI (n = 192)	Healthcare-associatedBSI (n = 85)	Community-onset BSI(n = 277)	Hospital-acquired BSI(n = 395)	p-value (ifsignificant)
Age (years) *	70 (IQR 60–81)	71(IQR 63–78)	70(IQR 61–80)	64(IQR 52–72)	p<0.001
Sex (f/m)	93/99	44/41	137/140	144/151	
BMI	26.27(IQR 23.55–29.30;n = 136)	25.22(IQR 22.31–28.08;n = 79)	25.82(IQR 22.83–28.55;n = 215)	25.02(22.11–28.91;n = 352)	
WBC (G/L)	11.94(IQR 8.23–16.9)	12.01(IQR 7.52–19.04)	11.96(IQR 8.15–17.46)	8.3(IQR 4.73–14.2;n = 127)	p<0.001
NLCR	0.88(IQR 0.81–0.92)	0.89(IQR 0.8–0.93)	0.88(IQR 0.81–0.92)	0.85(IQR 0.78–0.91;n = 86)	p = 0.036
C-reactive protein(mg/L)	141.5(IQR 51–242)	123.4(IQR 52.5–239.6;n = 84)	133.9(IQR 51.6–240;n = 276)	92.2(IQR 45.5–171.7;n = 388)	p<0.001
Procalcitonin(ng/mL)	1.27(IQR 0.39–8.28;n = 172)	3.92(IQR 0.66–19.46;n = 76)	1.62(IQR 0.42–12.03;n = 248)	0.89(IQR 0.27–5.19;n = 361)	p = 0.001
Interleukin-6(pg/mL)	493.8(IQR 154.9–2055;n = 155)	399.8(IQR 149.9–1188;n = 72)	442.3(IQR 153–1776;n = 227)	148(59.7–487.9;n = 332)	p<0.001
Serum creatinine(mg/dL)	1.32(IQR 1.01–2.06;n = 190)	1.74(IQR 1.16–3.18;n = 82)	1.36(IQR 1.03–2.42;n = 272)	1.24(IQR 0.84–2.33;n = 357)	p = 0.008
Days hospitalizedafter collection ofpositive bloodculture (days)	10 (IQR 3–16)	9 (IQR 6–15)	10 (IQR 5–16)	13 (IQR 8–23)	p<0.001
30-day mortality	16/192 (8%)	15/85 (18%)	31/277 (11%)	82/395 (21%)	p = 0.001
90-day mortality	18/192 (9%)	17/85 (20%)	35/277 (13%)	106/395 (27%)	p<0.001

* = Data available from all included patients unless otherwise stated.

Abbreviations: BMI, Body-mass index; BSI, blood stream infection; f, female; m, male; NLCR, neutrophyl leucocyte ratio; WBC, white blood cell count.

Causative pathogens detected in more than two patients (over all three BSI categories) are depicted in Table 2. The remaining 15 isolates (i.e. other gram-positive and gram-negative bacteria in Table 2) consisted of *Listeria* spp., *Bacteroides* spp., *Delftia acidovorans*, *Pasteurella multocis*, *Propionibacterium acnes*, *Marganella morganii, Agrobacterium tumefaciens, Actinomyces naeslundi, Bifidobacterium* sp., *Rothia dentocariosa, Micrococcus* sp., and *Comamonas acidovarans.* None of these organisms was isolated in more than two patients. In 35 patients a second pathogen and in ten a third was isolated from blood cultures. With regard to time-to-positivity 326/395 (83%) blood cultures in HA-, 75/85 (88%) in HCA-, and 174/192 (91%) in CA-BSI resulted positive on the same day or on the day after they had been obtained (within 48 hours).

**Table 2:** Causative pathogens in community-acquired and hospital-acquired BSI cases.

Causative pathogen	Community-acquired BSI(n = 192)	Healthcare-associatedBSI (n = 85)	Community-acquiredonset BSI(n = 277)	Hospital-acquired BSI(n = 395)	p-value (ifsignificant)
*Staphylococcus aureus*	25/192 (13%)	13/85 (15%)	38/277 (13.7%)	53/395 (13.4%)	
*Streptococcus* spp.	26/192 (14%)	12/85 (14%)	38/277 (13.7%)	20/395 (5.1%)	p<0.001
*Enterococcus* spp.	9/192 (5%)	5/85 (6%)	14/277 (5.1%)	49/395 (12.4%)	p = 0.001
Coagulase-negativeStaphylococci(>1 culture positive)	6/192 (3%)	7/85 (8%)	13/277 (4.7%)	74/395 (18.7%)	p<0.001
*Corynebacterium* spp.	0	1/85 (1%)	1/277 (0.4%)	3/395 (0.8%)	
Other gram-positive bacteria	4/192 (2%)	1/85 (1%)	5/277 (1.8%)	5/395 (1.3%)	
*Escherichia coli*	81/192 (42%)	28/85 (33%)	109/277 (39.4%)	65/395 (16.5%)	p<0.001
*Pseudomonas* spp.	4/192 (2%)	6/85 (7%)	10/277 (3.6%)	40/395 (10.1%)	p = 0.002
*Klebsiella* spp.	14/192 (7%)	9/85 (11%)	23/277 (8.3%)	36/395 (9.1%)	
*Enterobacter* spp.	3/192 (2%)	2/85 (2%)	5/277 (1.8%)	24/395 (6.1%)	p = 0.007
*Citrobacter spp.*	2/192 (1%)	2/85 (2%)	4/277 (1.4%)	4/395 (1%)	
*Salmonella spp.*	2/192 (1%)	2/85 (2%)	4/277 (1.4%)	0	
*Serratia spp.*	1/192 (1%)	2/85 (2%)	3/277 (1.1%)	8/395 (2%)	
*Proteus spp.*	3/192 (2%)	1/85 (1%)	4/277 (1.4%)	8/395 (2%)	
*Haemophilus influenza*	3/192 (2%)	0	3/277 (1.1%)	1/395 (0.3%)	
*Neisseria meningitidis*	3/192 (2%)	0	3/277 (1.1%)	0	
*Acinetobacter spp.*	0	2/85 (2%)	2/277 (0.7%)	4/395 (1%)	
*Stenotrophomonas spp.*	0	0	0	7/395 (1.8%)	
Other gram-negative bacteria	0	0	0	5/395 (1.3%)	
*Candida* spp.	0	0	0	32/395 (8.1%)	p<0.001

*Escherichia coli* followed by *Staphylococcus aureus* were the most frequently isolated pathogens. While *Streptococcus* spp. and *Escherichia coli* were isolated more frequently in those with community-onset BSI, *Enterococcus* spp., *Candida* spp., *Pseudomonas* spp., *Enterobacter* spp., and CoNS were isolated more frequently among those with HA-BSI. The analysis of CA- and HCA-BSI showed no significant differences. Also, the spectrum of pathogens did not differ markedly, although there was a trend towards fewer BSI due to *Escherichia coli* in the HCA-BSI group. Highest 30- and 90-day mortality rates were observed in cases of bacteremia due to *Pseudomonas* spp. (29% community-onset and 33% HA), followed by *Enterococcus* spp. (24% and 30%) and *Staphylococcus aureus* (20% and 29%), respectively.

Significantly more patients with HA-BSI had to be admitted to ICUs (122/395 [31%] HA-BSI vs. 34/277 [12%] community-onset BSI; p<0.001). Forty patients (median age 72 [IQR 62–79], 13 female, 27 male) died within 48 hours after collection of blood cultures (23 [5.8%] HA- and 17 [6.1%] community-onset BSI [9 with CA-BSI, 8 with HCA-BSI]). With regard to the outcome, 30-day (82/395 [21%] vs. 31/277 [11%]; p = 0.001) and 90-day overall mortality rates (106/395 [27%] vs. 35/277 [13%]; p<0.001) were significantly higher among patients with HA-BSI. HCA-BSI was associated with significantly higher 30-day (p = 0.04) and 90-day mortality rates (p = 0.02) when compared to CA-BSI.

Multivariable analyses were performed for prediction of 30-day and 90-day mortality. HA-BSI remained the strongest predictor of 30-day mortality (OR 2.64; 95% CI 1.43–4.88; p = 0.002). Other significant predictors were age (OR 1.02: 95% CI 1.00–1.04; p = 0.02) and IL-6 (OR 1.00; 95% CI 1.00-1.00; p = 0.021). Sex, CRP and body mass index were also included in the final model, but results were not significant. HA-BSI remained also the strongest predictor of 90-day mortality (OR 2.85; 95% CI 1.84–4.41; p<0.001). Other predictors were CRP (OR 1.00; 95% CI 1.00–1.01) and male sex (OR 1.65; 95% CI 1.09–2.5; p = 0.017). HCA-BSI was associated with significantly higher 30- (p = 0.04) and 90-day mortality rates (p = 0.02) when compared to CA-BSI.

Laboratory parameters are depicted in Table 1. Patients with community-onset BSI had significantly higher white blood cell counts (WBC), NLCR as well as CRP, PCT, IL-6 and creatinine levels. Significantly more patients with community-onset BSI had impaired renal function, whereas significantly less were neutropenic (neutrophil count <0.5 G/L; 15/277 [5.4%] community-onset BSI vs. 63/395 [15.9%] HA-BSI; p<0.001). Analysis of the differences between the subgroup of HCA-BSI and patients with CA-BSI showed that creatinine (1.74 mg/dL [IQR 1.16–3.18] vs. 1.32 mg/dL [IQR 1.01–2.06]; p = 0.002) and PCT (3.92 ng/mL [IQR 0.66–19.46] vs. 1.27 ng/mL [IQR 0.39–8.28]; p = 0.011) were significantly higher in those with HCA-BSI. Further, neutropenia occurred more frequently (11/85 [12.9%] vs. 4/192 [2.1%]; p<0.001) among patients with HCA-BSI. No differences for other parameters (including duration of hospitalization) were found.

## Discussion

Two main findings are evident. First, epidemiology of causative pathogens differed significantly between patients with community-onset and those with HA-BSI while no significant differences were found between patients with CA- and those with HCA-BSI. Second, mortality rates were significantly higher in patients with HA-BSI while levels of laboratory markers of inflammation were significantly higher in community-onset BSI.

In this study, *Escherichia coli was* the most frequently identified pathogen followed by *Staphylococcus aureus* and CoNS. *Streptococcus* spp. and *Escherichia coli* were isolated more frequently in patients with community-onset BSI, while *Enterococcus* spp., *Enterobacter* spp., *Candida* spp. (only isolated in patients with HA-BSI), *Pseudomonas* spp. and CoNS were isolated more frequently among those with HA-BSI. However, causative pathogens were comparable between patients with CA- and those with HCA-BSI. In a recent multicenter trial evaluating over 1400 patients with BSI, the three most common pathogens were *S. aureus* (n = 428, 28%), *E. coli* (n = 359, 24%), and CoNS (n = 148, 10%), though the type of infecting organism varied by location of acquisition [21]. As in our study *E. Coli* was the major pathogen identified in CA-BSI while CoNS were mainly identified in HA-BSI [21]. In that study, epidemiology of causative pathogens for HCA-BSI was found to differ markedly from HA- and CA-BSI. Compared to CA-BSI more *S. aureus*, CoNS and “other” pathogens and less *E. coli* and *Streptococcus* spp. were identified in the HCA-BSI group. Unfortunately, “other” pathogens were not further differentiated for the various locations of acquisition [21], [22]. Another study evaluating 821 BSI episodes in 15 hospitals in Spain reported that CoNS and *Enterococcus* spp. were significantly less frequent and *Streptococcus* spp. significantly more frequent in HCA-BSI when compared to HA-BSI [23]. Differences were also found between CA- and HA-BSI for *Streptococcus* spp. (more frequent in CA-BSI) and *Pseudomonas* spp. (more frequent in HA-BSI). With one exception, fungal BSIs were all acquired in hospital [23]. This finding is in line with our study where all 32 cases of candidemia were hospital-acquired.

HA-BSI was significantly associated with a worse outcome and remained the strongest predictor of mortality in multivariable analysis even though patients with community-onset BSI were significantly older than those with HA-BSI. Similar findings were reported by Diekema and colleagues who found a crude mortality of 34% associated with nosocomial BSI (compared to 14% in CA-BSI) [22]. In a recent study by Rodriguez-Bano and colleagues differences were less pronounced [23]. The overall 90-day mortality in BSI patients was found to be 21% in this study and was therefore slightly lower than in previous studies evaluating large patient cohorts one and two decades ago (23% and 24%) [22], [24].

HCA-BSI was associated with increased 30- and 90-day mortality rates when compared to CA-BSI. This is in accordance with previous studies [25], [26]. In another study healthcare acquisition of gram-negative BSI was also independently associated with higher 28-day (hazard ratio [HR] 3.73, 95% CI: 2.13–6.93) and 1-year mortality (HR 3.60, 95% CI: 2.57–5.15) when compared to acquisition in the community [27].

We further found that WBC as well as CRP, PCT and IL-6 were significantly lower in patients with HA- than in those with community-onset BSI. Same was found for NLCR which has been previously proposed by de Jager and colleagues as useful method for prediction of BSI [28], [29]. Significantly higher levels of laboratory markers for inflammation in patients with community-onset BSI might derive from a time delay between the onset of the first symptoms and the drawing of blood for laboratory analysis [30]. The fact that medical staff in emergency departments is usually reduced in after-hours might further extend the time to blood drawing in the vast majority of cases with community-onset BSI. It was shown previously that 27% of these patients arrived on a weekend and 58% in late hours. Many biomarkers, like CRP, are known to increase slowly in BSI, which may have resulted in lower levels in those patients for which blood cultures were obtained very early [31]. A PCT value of 0.1 ng/mL or less has recently been proposed as a useful marker to rule out bacteremia in the emergency department [32]. In our study the sensitivity of this cut-off for community-onset BSI was 96% and therefore comparable to previous studies while the sensitivity for HA-BSI was 92.9%. The sensitivity of IL-6 with the recommended cut-off of 10 pg/mL was even more promising. Only three cases had IL-6 values below this cut-off (sensitivity for the overall study population 99.6%). Previous studies have shown, however, that the specificity for these cut-offs, in particular for IL-6, is low [15], [32], [33].

The fact that overall 30- and 90-day mortality was chosen as primary endpoint may also be a limitation of the study as other factors than BSI may have contributed to mortality. In addition, inflammation markers were not available in all patients, and availability varied between e.g. CRP, PCT and IL-6. Consequently, comprehensive analysis of diagnostic and predictive potential of the markers was not possible. The primary objective of this study was therefore to report on epidemiology of BSI and differences between CA, HCA and HA acquisition and not to focus on biomarkers.

We conclude that causative pathogens differed markedly between community-onset and HA-BSI, while differences between CA- and HCA-BSI were by far less pronounced HA-BSI was associated with an increase in the risk of 30- and 90-day mortality when compared to community-onset BSI and therefore poses an important target for the most aggressive strategies for prevention and infection control.
